# Effects of Two Toxin-Producing Harmful Algae, *Alexandrium catenella* and *Dinophysis acuminata* (Dinophyceae), on Activity and Mortality of Larval Shellfish

**DOI:** 10.3390/toxins14050335

**Published:** 2022-05-10

**Authors:** Sarah K. D. Pease, Michael L. Brosnahan, Marta P. Sanderson, Juliette L. Smith

**Affiliations:** 1 Virginia Institute of Marine Science, William & Mary, P.O. Box 1346, Gloucester Point, VA 23062, USA; skpease@wm.edu (S.K.D.P.); mps@vims.edu (M.P.S.); 2 Woods Hole Oceanographic Institution, Redfield 3-30, MS 32, Woods Hole, MA 02543, USA; mbrosnahan@whoi.edu

**Keywords:** saxitoxin, okadaic acid, pectenotoxin, *Alexandrium catenella*, *Dinophysis acuminata*, *Crassostrea virginica*, harmful algae, harmful algal bloom, oyster larvae, shellfish

## Abstract

Harmful algal bloom (HAB) species *Alexandrium catenella* and *Dinophysis acuminata* are associated with paralytic shellfish poisoning (PSP) and diarrhetic shellfish poisoning (DSP) in humans, respectively. While PSP and DSP have been studied extensively, less is known about the effects of these HAB species or their associated toxins on shellfish. This study investigated *A. catenella* and *D. acuminata* toxicity in a larval oyster (*Crassostrea virginica*) bioassay. Larval activity and mortality were examined through 96-h laboratory exposures to live HAB cells (10–1000 cells/mL), cell lysates (1000 cells/mL equivalents), and purified toxins (10,000 cells/mL equivalents). Exposure to 1000 cells/mL live or lysed *D. acuminata* caused larval mortality (21.9 ± 7.0%, 10.2 ± 4.0%, respectively) while exposure to any tested cell concentration of live *A. catenella*, but not lysate, caused swimming arrest and/or mortality in >50% of larvae. Exposure to high concentrations of saxitoxin (STX) or okadaic acid (OA), toxins traditionally associated with PSP and DSP, respectively, had no effect on larval activity or mortality. In contrast, pectenotoxin-2 (PTX2) caused rapid larval mortality (49.6 ± 5.8% by 48 h) and completely immobilized larval oysters. The results indicate that the toxic effects of *A. catenella* and *D. acuminata* on shellfish are not linked to the primary toxins associated with PSP and DSP in humans, and that PTX2 is acutely toxic to larval oysters.

## 1. Introduction

*Alexandrium catenella*, associated with paralytic shellfish poisoning (PSP) and *Dinophysis acuminata*, associated with diarrhetic shellfish poisoning (DSP), are two common harmful algal bloom (HAB) species in the northeastern United States of America (USA). Regionally, cell concentrations of these two species are typically 1–100 cells/mL, but can exceed 1000 cells/mL, especially within subsurface thin layers and near bloom peaks [1,2,3]. *Alexandrium catenella* produces a suite of hydrophilic PSP toxins (PSTs), including saxitoxin (STX), while *D. acuminata* produces two classes of lipophilic toxins: the pectenotoxins (PTXs) and DSP toxins (DSTs)—consisting of okadaic acid (OA), dinophysistoxins (DTXs), and their derivatives. 

These species and their toxins may also threaten aquatic life. In shellfish, the most common vector of PSP and DSP to humans, these HAB species have wide-ranging effects that are sometimes, but not exclusively, related to human syndrome toxins. For instance, PSTs impair burrowing activity in some clam populations (*Mya arenaria* [4]) and interfere with the immunocompetence of oyster hemocytes (*Crassostrea gigas* [5]). PST-producing *Alexandrium* cells or their exudates reduce swimming activity and survival in many larval shellfish species [6,7,8]. Similarly, cultures of *A. catenella* cause adductor-muscle paralysis in adult oysters (*Crassostrea virginica* [9]), larval inactivity and a decrease in egg viability in pearl oysters (*Pinctada fucata martensii* [10,11]), and aberrant or arrested development and reduced growth, settling rates, and survival in larval oysters (*C. gigas* [12]). 

These cell and exudate effects are not always related to PST exposure. Non-PST bioactive compounds produced by *Alexandrium* species have been shown to reduce disease resistance in clams (*Ruditapes philippinarum* and *M. arenaria* [13]). Additionally, exposure of larval pearl oysters (*P. fucata martensii*) to non-PST-producing *Alexandrium affine* leads to larval inactivity [10]. Similarly, larval oysters (*C. gigas*) exhibit reduced swimming activity, feeding, growth, development, settlement yield, and malformations of the mantle on exposure to non-PST-producing *Alexandrium minutum* [14,15]. 

The effects on shellfish from *Dinophysis* species and/or associated toxins are comparable, though reports to date are more limited. Mussels (*Mytilus edulis*) exposed to OA exhibit reduced protein phosphatase activity and reduced larval viability [16,17], while PTX2 exposure to oysters (*C. gigas*) causes reduced fertilization success and larval mortality [18,19]. Likewise, exposure to *D. acuminata* causes oyster (*C. gigas*) oocyte mortality and reduced fertilization success, while gametes exposed to *Dinophysis sacculus* exhibit abnormal larval development [18]. 

Furthermore, co-occurrences and successive occurrences of *A. catenella* and *D. acuminata* blooms, as well as the co-occurrence of PSTs and DSTs, have been reported in Northport Bay, NY, USA [3]. These co-occurrences occurred at temperatures at which *C. virginica* in the region have been observed to spawn [20]. The potential combined effects of these HAB species or their toxins on bivalves have not been studied; however, a recent study that explored the individual and combined effects of *A. catenella* and *D. acuminata* on two larval, estuarine finfishes found reduced growth and swimming activity on exposure to either HAB species [21]. Lethality in the latter study was associated with exposure to *A. catenella* alone, with no additive effects through co-exposure with *D. acuminata*. 

A goal of this study was to explore the effects of these HAB species and toxins—traditionally associated with human health issues—on shellfish health, and to assess whether effects were due to PSTs and/or DSTs, or other modes of toxicity. We assessed the individual and combined effects of *A. catenella* and *D. acuminata* and their toxins on the survival and swimming activity of oyster larvae (*C. virginica*). These animals were too small to consume *A. catenella* or *D. acuminata* directly, so all assays evaluated non-feeding interactions with live HAB cells, suspended particles, extracellular toxins and bioactive compounds, depending on the bioassay. Bioassays included 96-h exposures to: (1) *A. catenella* and *D. acuminata* live cells, administered individually; (2) *A. catenella* and *D. acuminata* lysates, administered individually and combined; and (3) pure toxins from *A. catenella* (STX) and *D. acuminata* (OA and PTX2), administered individually and combined (Table 1). Co-exposures were limited to toxin and lysate experiments to avoid complications that might have arisen from interactions between the two live HAB species.

## 2. Results

### 2.1. Larval Inactivity and Mortality

#### 2.1.1. Live-Cell Bioassay

**Figure 1:**
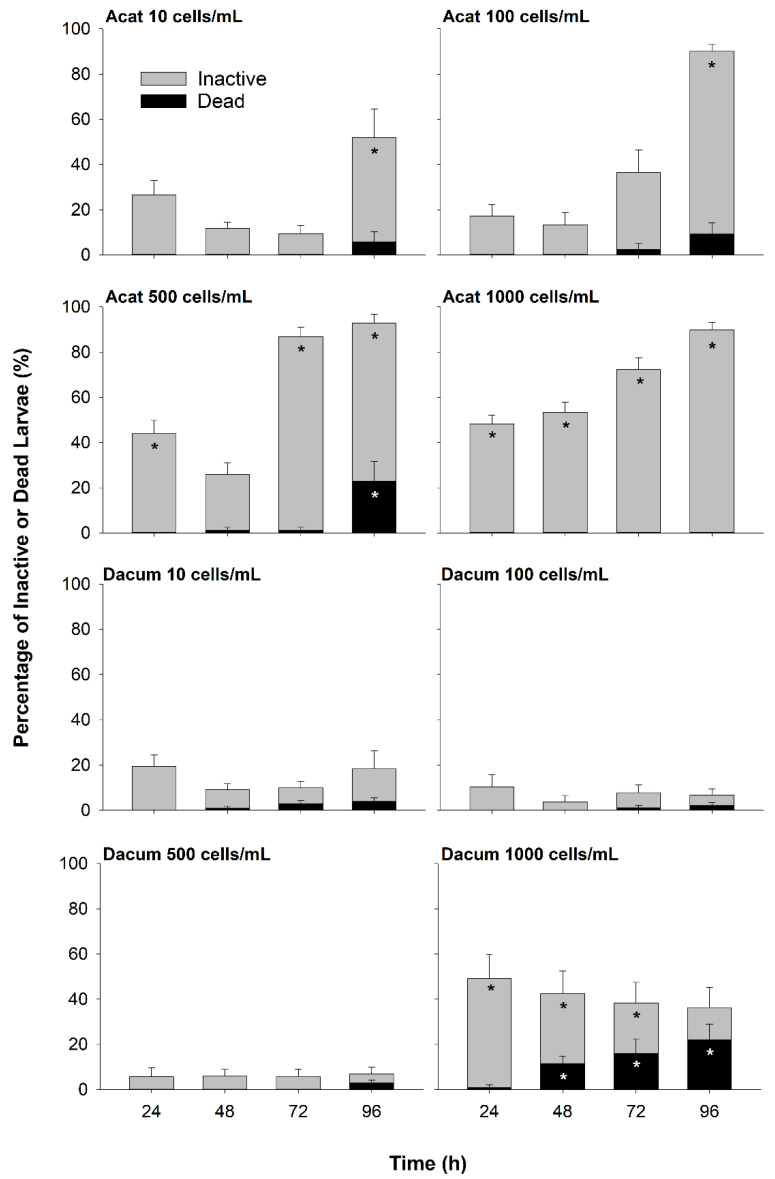
Percentage of inactive and dead larvae over time (h) in the live-cell bioassay, when larval oysters were exposed to *Alexandrium catenella* (Acat) or *Dinophysis acuminata* (Dacum), at four different initial cell concentrations. Error bars show the standard error (*n* = 10 wells per treatment). Data points that were significantly different from the Fed and Unfed treatments are denoted by an asterisk.

Depression of larval swimming activity, i.e., inactivity including death, was observed in the higher cell concentration treatments of live-cell *A. catenella* and *D. acuminata*. By 96 h, all of the *A. catenella* treatments had significantly higher larval inactivity than the Fed and Unfed treatments (Fed: 11.6 ± 4.0%, Unfed: 5.7 ± 2.5%, Acat_10_: 51.9 ± 12.7%, Acat_100_: 90.1 ± 3.0%, Acat_500_: 92.8 ± 4.0%, Acat_1000_: 89.8 ± 3.4% standard error [*SE*], *n* = 10 wells per treatment, Tukey–Bonferroni: all *p* < 0.0125; Figure 1, Table S1). Additionally, larvae exposed to the Acat_500_ treatment exhibited moderate mortality (22.8 ± 8.7% *SE*; Figure 1), significantly higher than the Fed and Unfed treatments (Fed: 0.9 ± 0.9% *SE*, Unfed: 0%, Tukey–Bonferroni: all *p* < 0.0125), while the Acat_10_, Acat_100_, and Acat_1000_, treatments exhibited low mortality (Acat_10_: 5.7 ± 4.6%; Acat_100_: 9.2 ± 5.0% *SE*; Acat_1000_: 0%; Figure 1, Table S2). With *D. acuminata*, larval inactivity and mortality were only significant in the Dacum_1000_ treatment, with inactivity peaking at 24 h (49.2 ± 10.6% *SE*; Figure 1, Table S1). By 48 h, the Dacum_1000_ treatment had significantly higher mortality than the Fed and Unfed treatments, peaking at 96 h (21.9 ± 7.0% *SE*, Tukey–Bonferroni: all *p* < 0.0125; Figure 1, Table S2). Significant predictors in the models of larval inactivity and mortality included time, treatment, and their interaction (full model outputs in Tables S3 and S4, respectively). Cell concentrations of *A. catenella* and *D. acuminata* stayed the same or increased from 0 to 96 h across all treatments (Figure S1).

#### 2.1.2. Lysate Bioassay

**Figure 2:**
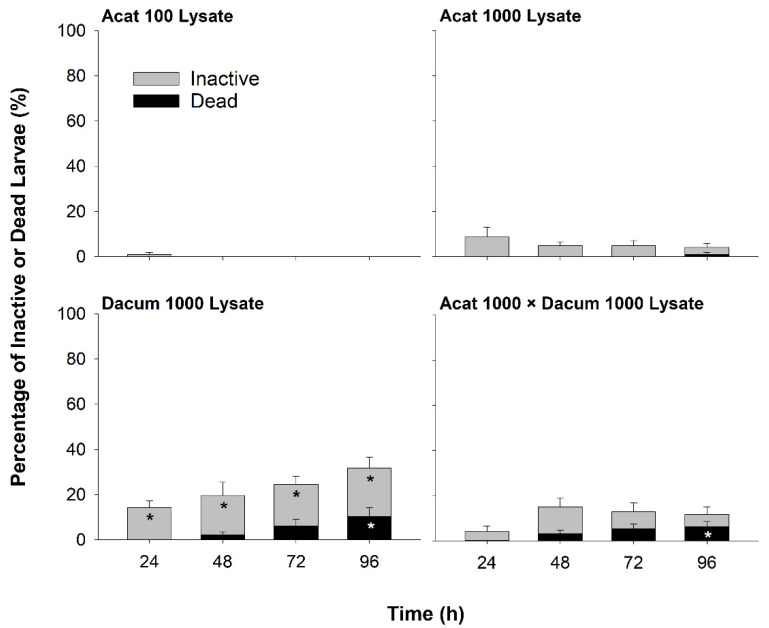
Percentage of inactive and dead larvae over time (h) in the lysate bioassay, when larval oysters were exposed to *Alexandrium catenella* (Acat) and *Dinophysis acuminata* (Dacum) lysate treatments. Numbers represent cell concentration equivalents (cells/mL equiv.). Error bars show the standard error (*n* = 10 wells per treatment). Data points that were significantly different from the Fed and Unfed treatments are denoted by an asterisk.

Effects on larval oysters from exposure to lysates were generally lower than from live-cell exposure (Figure 1 and Figure 2). Lysates of *A. catenella* had no significant effects while *D. acuminata* lysate exposure caused significant larval inactivity compared to Fed and Unfed treatments at all timepoints, peaking at 96 h (Dacum_1000_: 31.8 ± 4.8%, Fed: 2.0 ± 1.4%, Unfed: 2.9 ± 1.5% *SE*, Tukey–Bonferroni: all *p* < 0.0125; Figure 2, Table S5). Mixture of *D. acuminata* and *A. catenella* lysates moderated the effect of *D. acuminata* lysate exposure (Figure 2), but both the Acat_1000_ × Dacum_1000_ and Dacum_1000_ lysate treatments caused low to moderate mortality by 96 h, significantly higher than Fed or Unfed treatments (Acat_1000_ × Dacum_1000_: 6.2 ± 2.3%, Dacum_1000_: 10.2 ± 4.0%, Fed: 0%, Unfed: 2.0 ± 1.3% *SE*, Tukey–Bonferroni: all *p* < 0.0125). No other lysate treatments produced significant mortality in this set of experiments (Acat_100_: 0%, Acat_1000_: 0.9 ± 0.9% *SE*, Tukey–Bonferroni: all *p* > 0.0125; Figure 2, Table S6). Significant predictors in the model of larval inactivity included treatment and the interaction between treatment and time (full model output in Table S7). Significant predictors in the model of larval mortality included time, treatment, and their interaction (full model output in Table S8).

#### 2.1.3. Pure Toxin Bioassay

**Figure 3:**
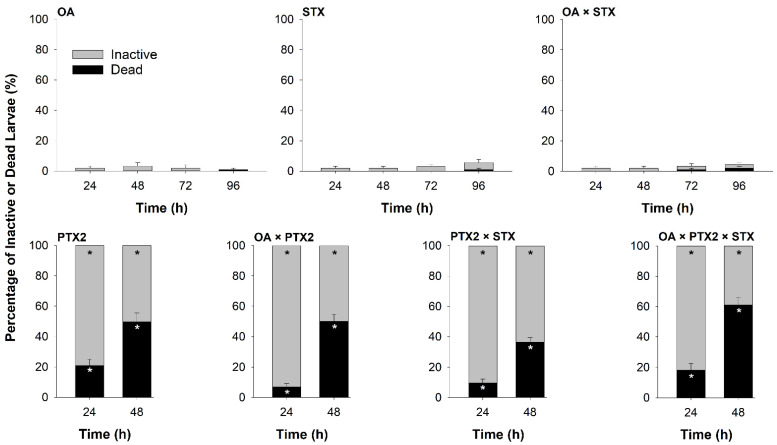
Percentage of inactive and dead larvae over time (h) in the pure toxin bioassay, when larval oysters were exposed to okadaic acid (OA), pectenotoxin-2 (PTX2), and saxitoxin (STX), alone or in combination at toxin concentrations equivalent to 10,000 cells/mL. Treatments PTX2, OA × PTX2, PTX2 × STX, and OA × PTX2 × STX were terminated at 48 h to collect toxin samples. Error bars show the standard error (*n* = 10 wells per treatment). Data points that were significantly different from the Carrier treatment are denoted by an asterisk.

Among the three pure toxins tested (STX, OA, and PTX2), only PTX2 caused severe inactivity and mortality. All larval oysters exposed to pure PTX2 were inactive within 24 h and experiments were therefore terminated after 48 h (i.e., 2 days earlier than the other single toxin treatments evaluated; Figure 3). All treatments that included PTX2 exhibited significantly higher mortality than the Carrier treatment at both 24 h (Kruskal–Wallis: χ^2^ = 47, df = 7, *p* < 0.0001, Dunn tests: all *p* < 0.05) and at 48 h (Kruskal–Wallis: χ^2^ = 71, df = 7, *p* < 0.0001, Dunn tests: all *p* < 0.05). Highest mortality was observed on exposure to all three toxins (OA × PTX2 × STX: 61.1 ± 4.9% *SE*) but PTX2 alone and other pairwise combinations produced similar levels of larval death (PTX2: 49.6 ± 5.8%, OA × PTX2: 50.0 ± 4.7%, PTX2 × STX: 36.5 ± 2.8% *SE*; Figure 3).

In contrast, larval inactivity over the 96 h in the OA, STX, and OA × STX treatments was low (all < 6%; Figure 3), and not significantly different from the Carrier treatment (Carrier: 1.0 ± 1.0% *SE*, full model output in Table S9). Similarly, larvae from the OA, STX, and OA × STX treatments all exhibited very low mortality by 96 h (OA: 1.0 ± 1.0%, STX: 1.0 ± 1.0%, OA × STX: 2.0 ± 1.3%, Carrier: 1.0 ± 1.0% *SE*; Figure 3; full model output in Table S10).

### 2.2. Toxins in HAB Cultures, Water, and Oysters

Initial hatchery water used in the bioassays did not contain quantifiable toxin concentrations. Trace levels of PTX2, the DST dinophysistoxin-1 (DTX1), and the PSTs gonyautoxin 2 (GTX2) and decarbamoylneosaxitoxin (dcNEO) were detected but not quantifiable (<LOQ) in various lots of initial hatchery water, and were therefore negligible compared to concentrations used in these experiments.

The toxin profile for the *A. catenella* culture was dominated by the STX congener C2, with 3.9 pg C2/cell (>90%); STX and GTX3 were also detected but were <LOQ. The extracellular fraction of the *A. catenella* culture contained trace (<LOQ) amounts of dcNEO. Lysate from *A. catenella* had trace (<LOQ) amounts of dcNEO and GTX5. Samples for the analysis of intracellular PSTs in *A. catenella* culture were pelleted and aspirated to concentrate toxins prior to quantification, improving their associated detection limit (DL). Inclusion of a PST concentration step in extractions from extracellular toxin samples (media and lysate) would likely have improved quantification of PSTs from these fractions but was not attempted in this study. 

The toxin profile for the *D. acuminata* culture was dominated by PTX2. Intracellular toxins included 8.0 pg PTX2/cell (91%), 0.4 pg OA/cell (5%), and 0.2 pg DTX1/cell (2%), while only PTX2 was quantifiable (0.2 pg PTX2/cell, 2%) in the extracellular fraction, with trace (<LOQ) amounts of OA (Figure S2). Lysate from *D. acuminata* had a similar toxin profile to the culture, with 4.6 pg PTX2/cell equiv. (90%), 0.3 pg OA/cell equiv. (6%), 0.2 pg DTX1/cell equiv. (4%; Figure S2). 

Larval oysters in all of the bioassays bioaccumulated PTX2 (Table 2). In the live-cell bioassay, oysters bioaccumulated increasing amounts of PTX2 with exposure to increasing *D. acuminata* concentration. Oysters exposed to the highest concentration of live or lysed *D. acuminata*, 1000 cells/mL or equivalent, accumulated similar PTX2 concentrations (5.2 and 3.7 pg PTX2/oyster, respectively). Oysters exposed to concentrations of pure PTX2, equivalent to 10,000 cells/mL, accumulated an order of magnitude more PTX2 (40.2–50.0 pg PTX2/oyster) than oysters exposed to 1000 *D. acuminata* cell equivalents/mL of live cells or lysate. PTX2 was below the detection limit (<DL) in control oysters. Trace amounts of OA and DTX1 (<LOQ) were occasionally detected in larvae.

In the pure toxin bioassay, PTX2 in well water samples declined over 48 h, with or without oysters present, from 70.7 ± 14.5 standard deviation (*SD*) ng PTX2/mL at the start of treatment to 28.1 ± 3.7 *SD* ng PTX2/mL without oysters, and to 34.7 ± 5.9 *SD* ng PTX2/mL with oysters. Trace amounts of OA and DTX1 (<LOQ) were occasionally detected in well water samples as well.

**Table 2:** Oyster bioaccumulation of toxins associated with *Dinophysis acuminata*.

Bioassay	Treatments ^a^	Time Collected (h)	Oysters per Sample ^b^	Toxin (pg/oyster) ^c^		
				OA	DTX1	PTX2
N/A	Control	0	70 *	<DL	<LOQ	<DL
Live-Cell	Dacum 10	96	80	<DL	<LOQ	<LOQ
	Dacum 100	96	69	<DL	<DL	<LOQ
	Dacum 500	96	72	<LOQ	<DL	1.8
	Dacum 1000	96	70	<DL	<DL	5.2
Lysate	Dacum 1000	96	68	<LOQ	<LOQ	3.7
	Acat 1000 × Dacum 1000	96	65	<DL	<LOQ	2.5
Pure Toxin	PTX2	48	74	<DL	<DL	40.2
	OA × PTX2	48	70	<DL	<DL	45.3
	PTX2 × STX	48	71	<DL	<DL	50.0
	OA × PTX2 × STX	48	70	<DL	<DL	47.9

N/A = not applicable. Oysters were pooled into one sample per treatment and results were normalized to pg toxin/oyster. ^a^ Acat = *Alexandrium catenella*, Dacum = *Dinophysis acuminata,* DTX1 = dinophysistoxin-1, OA = okadaic acid, PTX2 = pectenotoxin-2, and STX = saxitoxin; numbers represent cell concentrations or cell concentration equivalents (cells/mL or cells/mL equiv.). ^b^ Direct counts of oysters were performed by microscope, unless otherwise noted. ^c^ <DL = below detection limit; <LOQ = below limit of quantitation. * Oysters per sample was estimated volumetrically. Counts from samples of concentrated larvae in hatchery water provided an estimate of oysters/mL, allowing the calculation of the volume needed to sample approximately 70 oysters.

## 3. Discussion

This is the first study to assess the effects of *A. catenella*, *D. acuminata*, OA, PTX2, and STX on larval eastern oysters (*C. virginica*), and to test combined effects of *A. catenella* and *D. acuminata* lysates and toxins on a bivalve. Overall, STX- and OA-only exposures were not toxic to oysters, but PTX2, live *A. catenella*, and live or lysed *D. acuminata* all produced larval inactivity and death. This suggests that non-syndrome-related bioactive compounds or toxins produced by these dinoflagellates, i.e., PTX2, are toxic to larval oysters. Mortality was highest in oysters exposed to pure PTX2 (10,000 cells/mL equiv.), reaching ~50% by 48 h, followed by oysters exposed to live *A. catenella* or *D. acuminata* (500 or 1000 cells/mL, respectively) that both suffered > 20% mortality by 96 h. Larvae that did not die were rendered inactive by 24 h when exposed to pure PTX2. Similarly, 24-h exposure to 1000 cells/mL of either live *A. catenella* or *D. acuminata* caused larval inactivity, but to a lesser extent. The demonstrated toxicity of PTX2 to larval oysters, along with the observed mortality during exposure to a high cell concentration of live *D. acuminata* (1000 cells/mL), suggests that concentrated blooms of PTX2-producing *D. acuminata* could stress larval oysters. 

### 3.1. Effects of A. catenella on Larval Oysters

In this study, larval oysters exposed to live *A. catenella* experienced some mortality and significant inactivity by 96 h, while *A. catenella* lysate and pure STX had no measured effect. Inactivity was commonly observed among larvae by the end of the 96-h bioassay in response to exposure to live *A. catenella* across all cell concentrations tested (10–1000 cells/mL; Figure 1). The highest cell concentration (1000 cells/mL) resulted in significant inactivity of larvae throughout the 96-h bioassay. These findings agree with previous studies that exposed larval oysters to PST-producing and non-PST-producing strains of *Alexandrium* spp., demonstrating that PSTs did not play a role in the observed inactivity [11,15]. 

Though inactivity was common, mortality associated with *A. catenella* exposure, if it occurred at all, was modest relative to PTX2. The only live-cell *A. catenella* treatment to exhibit significant larval mortality was exposure to 500 cells/mL for 96 h, a result that was preceded by a sharp rise in inactivity at 72 h (Figure 1). It is unlikely that larval deaths in this treatment were due to physical interactions between the oysters and *A. catenella* because no deaths occurred at the higher cell concentration tested (1000 cells/mL). Oyster larvae also did not ingest *A. catenella* (Figure S1), as these cells are 3X the size of particles ingested by oysters at this life stage [22,23]. This suggests that larval oysters interacted with extracellular toxins or bioactive compounds produced by *A. catenella*. Exposure of larvae most likely occurred through exposed tissues, e.g., gill, mantle, velum, and/or digestive epithelium. 

In contrast to treatments with live cells, neither larval inactivity nor mortality occurred in oysters exposed to *A. catenella* lysate or to pure STX. In the pure toxin bioassay, larvae were exposed to a high concentration of purified STX (27 ng STX/mL) that was equivalent to 10,000 cells/mL of *A. catenella*, representing a concentration approximately 2–3-fold higher than the most extreme bloom conditions observed in the New England region, USA [1]. The lack of STX effects observed in this study is consistent with work by Yan and colleagues [6], who found that STX exposure to scallop eggs, even at extreme doses (5900 ng STX/mL), did not replicate the toxicity of live *Alexandrium tamarense.* STX and other PSTs may not be toxic to these animals, or may require ingestion for deleterious effects to occur. Both this and the Yan et al. [6] study exposed animals only to dissolved toxins, which may not accumulate or be absorbed in sufficient amounts to affect early life stages.

These results are in contrast to other work that has demonstrated STX uptake and toxicity in non-feeding fish larvae and embryos [24,25,26]. Several studies have also implicated PSTs more generally with negative effects on adult shellfish (reviewed in [27]). In the current study, dissolved STX and cell lysate were not toxic to larval oysters, while live *A. catenella* cells were. Together, the results indicate that other bioactive compounds were responsible for the observed deleterious effects, and that their production required co-incubation of *A. catenella* with its potential grazer. This theory was also proposed to explain toxicity in scallops (*P. maximus*) and another species of oyster (*C. gigas*) exposed to a non-PST-producing strain of *A. minutum* [15,28,29]. Various bioactive compounds have been proposed as the cause of this type of *Alexandrium* toxicity, including lytic compounds, reactive oxygen species (ROS), and polyunsaturated fatty acids (PUFAs) [6,8,11,15,29,30,31,32,33,34,35]. Further research is needed to identify the non-PST bioactive compounds produced by *Alexandrium* spp. and their mechanisms of toxicity on aquatic life. 

### 3.2. Effects of D. acuminata on Larval Oysters

Larval oysters exposed to live or lysed *D. acuminata*, or pure PTX2, experienced significant mortality and inactivity, while exposure to pure OA had no measured effect. Oysters exhibited significant inactivity at 24 h in the 1000 cells/mL live *D. acuminata* treatment and larval mortality increased with time and was significantly different from Fed or Unfed treatments from 48 h on (21.9% by 96 h; Figure 1). As with *A. catenella*, toxicity may be linked to extracellular compounds produced by this species, as *D. acuminata* is too large for oyster larvae to ingest and cells were not depleted during the bioassay (Figure S1 [22,36]). Larval oysters bioaccumulated PTX2 in both live-cell and lysate treatments (Table 2). However, live-cell effects were only significant in the highest concentration tested (1000 cells/mL), which is rarely observed in nature [3]. Such intense *D. acuminata* blooms likely stress larval oysters where and when they co-occur. 

Mortality and inactivity from *D. acuminata* lysate were both significant by 96 h, but effects were approximately half that of an equivalent concentration of live *D. acuminata*. The reduced toxicity of lysate was mirrored by lower PTX2 concentration in the lysate (Figure S2). The sharp decline in PTX2 concentration during pure toxin incubations without oysters (60% over 48 h, pH 7.73, see Section 2.2) suggests that PTX2 likely broke down quickly during lysate preparation and in experimental treatments. In contrast, OA and DTX1 intracellular concentrations were similar to that of lysate, suggesting these toxins were more stable. Extracellular PTX2 may rapidly hydrolyze to less-toxic seco acids [37], while in the live-cell treatments, cells may actively produce and release PTX2 throughout the bioassay. Alternatively, PTX2 may have been differentially lost through surface adsorption to assay wells, though this effect should have been minimized in the current study by the use of glass inserts (see Section 5.1). 

When exposed to PTX2 (179 ng/mL), larvae suffered rapid mortality (~50% by 48 h) and complete (100%) inactivity when compared to the Carrier treatment or any OA treatments. This suggests that PTX2 played a significant role in larval mortality and inactivity in the *D. acuminata* live-cell and lysate exposures. In a similar study using larval fish (*Cyprinodon variegatus*), exposure to pure PTX2 (0.0003–0.005 ng/mL) led to significant gill pathology and mortality by 96 h [19]. In that same study, live cells and lysate from a strain of *D. acuminata* that produced primarily PTX2 and some OA and DTX1, did not cause any mortality; however, exposure to live cells caused some gill pathology. In the current study, a PTX2 concentration of 0.0003 ng/mL could have been produced by 0.0375 cells/mL with the strain of *D. acuminata* used (DATC03, 8.0 pg PTX2/cell, Figure S2). This is a low cell concentration that is observed in regions where *D. acuminata* occurs [3], suggesting the possibility of toxic effects of PTX2 on aquatic life in the field. 

Unlike PTX2, OA by itself was not acutely toxic to larval oysters. Oysters exposed to OA exhibited low inactivity (<4%) and mortality (1%) throughout the 96 h of the larval assays. These results corroborate those of a similar study that found no effect of pure OA (0.00006 ng/mL) on oyster gametes (*C. gigas* [18]). Larger OA doses than tested in this study (5.4 ng/mL) have been reported to produce toxic effects on aquatic life. OA at concentrations at or above 37.8 ng/mL reduced viability of larval mussels (*M. edulis* [16]), and concentrations of 3000–8000 ng/mL were acutely toxic to larval zebrafish (*Danio rerio* [38]). To achieve comparable levels, the *D. acuminata* strain used for these experiments (DATC03) would need to reach concentration of nearly 100M cells/L (Figure S2), suggesting toxic effects on aquatic life from *D. acuminata* populations similar to this strain are unlikely. 

Toxin profiles of *D. acuminata* strains from around the world vary [39,40]; the strain used in this study (DATC03) primarily produced PTX2, but also produced OA and DTX1 (Figure S2). The majority of PTX2 and OA, and all of the DTX1, were intracellular in the *D. acuminata* culture. Tong and colleagues [40] have suggested that northwestern Atlantic *D. acuminata* strains, such as DATC03, have lower DST content than *D. acuminata* from other regions of the world. The results presented here suggest that any reduced risk of DSP from northwestern Atlantic *D. acuminata* may come with a tradeoff in that their higher PTX2 levels can pose a greater threat to shellfish and other aquatic life.

### 3.3. Potential Effects of Co-Exposure to A. catenella and D. acuminata

*Dinophysis acuminata* lysate and PTX2 were responsible for the larval mortality and inactivity observed in co-exposures of lysates and pure toxins, respectively. Co-exposure to OA and STX had no measured effect on larval oysters, with no mortality and low (2%) inactivity by 48 h. Co-exposure of PTX2 with OA and/or STX, however, did not alter the acute toxicity of PTX2 to larval oysters. PTX2 alone, or in combination with OA and/or STX, led to complete (100%) larval inactivity and 37–61% mortality by 48 h (Figure 3). Furthermore, the addition of *A. catenella* lysate reduced the negative effects of *D. acuminata* lysate on larval activity, but significant larval oyster mortality associated with exposure to *D. acuminata* lysate was not changed by the addition of *A. catenella* lysate. In contrast, Rountos et al. [21] found that *A. catenella* drove toxicity to larval fish, not *D. acuminata*, with a combined treatment of the live HAB species showing similar toxicity to *A. catenella* alone. These contrasting results may indicate fundamental differences in the toxicity of these HAB species to fish (vertebrates) compared to bivalves (invertebrates) or might be related to variations in exposure routes (live cells versus lysates in combined treatments) or HAB strain differences. Studies of combined effects (*in vivo* or *in vitro*) of HAB toxins are still rare (reviewed in [41]), and similar studies assessing the combined effects of pure STX with OA and/or PTX2 are not available for comparison. Given the complex nature and sometimes concurrent or sequential occurrence of HAB species, co-exposure studies offer valuable insights into the effects of HAB and HAB toxin exposures in the environment. 

## 4. Conclusions

*Alexandrium* and *Dinophysis* spp. are monitored in the field primarily to protect human health from PSP and DSP, respectively. Shellfish, the vectors of these syndromes, can also be negatively impacted by these HAB species. In this study, larval oysters exposed to live *A. catenella* exhibited swimming arrest and some mortality. Additionally, PTX2, a toxin produced by *D. acuminata*, was acutely toxic to larval oysters, causing rapid swimming arrest and mortality. The primary toxins associated with PSP and DSP (STX and OA, respectively) were not acutely toxic to larval oysters in this study. These HAB species can produce suites of bioactive compounds capable of affecting both shellfish and humans. 

## 5. Materials and Methods

### 5.1. Experimental Design

A series of 96-h, static bioassays (live HAB cell, HAB cell lysate, and pure toxin) were performed in the laboratory to assess acute effects of *A. catenella* and *D. acuminata* and their toxins on larval oysters (Table 1). Bioassays were performed in 24-well tissue culture plates (Falcon^®^, Corning Inc., Corning, New York, NY, USA), with ten replicate wells per treatment. Treatments were made by diluting live algal culture (live-cell bioassay), lysed algal culture (lysate bioassay), or purified toxin (pure toxin bioassay), with treated hatchery water [42] to reach desired concentrations. Lysate treatments were tested to assess the effects of extracellular, as well as intracellular, bioactive compounds and toxins associated with these HAB species alone and in combination. Additionally, the lysate bioassay facilitated assessment of whether toxic effects required living cells. Pure toxin treatments were tested to assess the effects of STX, OA, and PTX2 toxins alone and in combination in the absence of cell effects or other bioactive compounds associated with these HAB species. 

Wells were loaded with 1 mL of treatment before approximately ten, actively swimming, seven-day-old, larval oysters were added. During the bioassays, well plates were stored in a Percival AL36L4 incubator (Percival Scientific, Perry, IA, USA; 19 ± 0.5 *SD* °C, 38 ± 10 *SD* µmol/m^2^/s, 14:10 h light–dark cycle). Well plate lids were removed only during daily observations of larval oyster activity and mortality (see Section 5.2). Throughout the bioassay, no water changes were performed, no algal additions were made, and no larval oysters were removed. To reduce adsorption of lipophilic toxins to plastic well plates, 1-mL glass microbeaker inserts (Electron Microscopy Sciences, Hatfield, PA, USA) were used in all wells. 

#### 5.1.1. Live-Cell Bioassay

In the live-cell bioassay, treatments consisted of each HAB species, *A. catenella* or *D. acuminata*, at the following cell concentrations: 10, 100, 500, and 1000 cells/mL (treatment abbreviations provided in Table 1). Cell concentrations were selected to test acute toxicity of these HAB species to larval oysters. Other treatments included an Unfed treatment, i.e., no algae present, to account for changes in oyster activity or mortality due to malnourishment, and a Fed treatment, in which oysters received 25,000 cells/mL of *Pavlova pinguis* (Pavlovophyceae; see Section 5.4). 

Changes in HAB cell concentrations throughout the live-cell bioassay were monitored using additional HAB treatment wells. Daily, triplicate wells of each treatment level of *A. catenella* and *D. acuminata* were collected, fixed with 10% neutral buffered formalin (Pharmco-Aaper, Brookfield, CT, USA), and stored at 4 °C until they could be counted. Samples were enumerated in a Sedgewick–Rafter slide using light microscopy (Olympus CX31 and CX41, Olympus Corp., Shinjuku, Tokyo, Japan). Samples of each HAB culture (equivalent to 20,000 cells) were collected, separated into intracellular and extracellular components by gentle centrifugation: 12 min at 3234× *g* at 4 °C (5804R, Eppendorf, Hauppauge, New York, NY, USA), and frozen at −20 °C, for toxin extraction and analysis of endogenous PSTs and DSTs. 

#### 5.1.2. Lysate Bioassay

In the lysate bioassay, *A. catenella* and *D. acuminata* cultures were lysed and diluted with hatchery water to create treatments that were equivalent to cell concentrations used in the live-cell bioassay. Treatments included two *A. catenella* lysate treatments (100 and 1000 cells/mL equiv.), one *D. acuminata* lysate treatment (1000 cells/mL equiv.), and one lysate co-exposure treatment representing 1000 cells/mL equiv. of both HAB species. Unfed and Fed treatments, as described in Section 5.1.1, were also included. To attempt to capture the range of effects of oyster larvae exposure to *A. catenella* lysate, since *A. catenella* live-cell treatments did not show a typical dose–response relationship, two treatment levels of *A. catenella* lysate were tested (100 and 1000 cells/mL equiv.). 

To lyse the cultures, two days prior to the start of the bioassay, both HAB cultures were sieved through 10 µm Nitex mesh, resuspended in hatchery water, and enumerated. Cultures were bath sonified for 15 min at 40 kHz (M5800H, Branson, Danbury, CT, USA), frozen and thawed 3X, and probe sonified on ice (Digital Sonifier-450, Branson, Danbury, CT, USA) for 10–20 min in 20 s cycles at 40% amplitude. Cell lysis was verified using light microscopy (Olympus CX31 and CX41, Olympus Corp., Shinjuku, Tokyo, Japan). Lysate was stored at −20 °C until ready for use in the bioassay, and a portion (equivalent to 8825 *A. catenella* and 20,150 *D. acuminata* cells) was aliquoted for toxin extraction and analysis, and frozen at −20 °C.

#### 5.1.3. Pure Toxin Bioassay

To assess linkage of live-cell and lysate toxic effects to HAB toxins STX, OA, and PTX2, oyster larvae were exposed to high levels of purified toxins. As a proof-of-concept, a simplified toxin profile was administered based on intracellular toxin quotas from isolates of *A. catenella* (Salt Pond isolate: 2.7 pg STX/cell) and *D. acuminata* (DATC03: 0.54 pg OA/cell, 17.9 pg PTX2/cell) from Nauset Marsh (NM; Cape Cod, MA USA). Final concentrations of toxins were made to represent 10,000 cells/mL equiv. for each HAB species. Certified toxin reference materials purchased from the National Research Council Canada (NRC CRM-STX-f, NRC CRM-OA-d, NRC CRM-PTX2-b) were used in making the treatment levels: 27 ng STX/mL, 5.4 ng OA/mL, and 179 ng PTX2/mL. All three toxins were administered in a full factorial design (Table 1). A combined Carrier control treatment was included in the pure toxin bioassay: 4% methanol (MeOH) and 3 µM hydrochloric acid (HCl), based on the OA × PTX2 × STX treatment. 

Changes in PTX2 concentration during the pure toxin bioassay were monitored using additional pure toxin treatment wells without oysters. Triplicate well water samples were collected at the bioassay start and termination timepoints for toxin analysis (see Section 5.3).

### 5.2. Larval Oyster Metrics and Well Water

During the 96-h bioassays, well plates were removed daily from the incubator for assessment of larval oyster mortality and activity by light microscopy (Olympus CKX53 or IX50 inverted microscopes, Olympus Corp., Shinjuku, Tokyo, Japan). An oyster was counted as *dead* if it exhibited no ciliary movement or had intact and empty shells; observations were made at 40× magnification. At the end of each bioassay, well plates were briefly placed in a −20 °C freezer to cause larvae to stop swimming and fall to the bottom of the wells, allowing for a *total* larval count of each well. To calculate mortality at each timepoint for each well, the following equation was used: *% dead =* *(dead/total)* × *100*. The average *% dead* of larvae at each timepoint for each treatment across 10 replicate wells was then calculated. 

Larval activity in each well was assessed every 24 h [6,11,42]. Briefly, plates were gently swirled to cause larvae to stop swimming and sink to the bottom of the wells. Swimming naturally resumed, and after 5 min, the number of non-swimming, *inactive* larvae in each well was recorded. *Inactive* oyster counts included dead larvae. To calculate the percentage of *inactive* larvae in each well at each timepoint, the following equation was used: *% inactive = (inactive/total)* × *100*. The average *% inactive* of larvae at each timepoint for each treatment across 10 replicate wells was then calculated. 

Larval oysters were collected during the three bioassays for the quantification of DSTs and PTXs. At the end of the bioassays, larvae were pooled by treatment to reach biomass requirements for toxin analysis (7 wells pooled ≈ 70 oysters); toxin results were normalized to pg toxin/oyster. Larvae were collected on 64 µm Nitex mesh, rinsed with hatchery water, with excess water removed via aspiration. An additional control sample of pooled oysters was collected at the start of the bioassays to assess background toxin concentrations. A pipette tip fitted with 64 µm Nitex mesh was used to collect well water without oysters from triplicate wells in both of the pure PTX2 treatments, the treatment with oysters, and the treatment without oysters (see Section 5.1.3). PST concentrations were not measured in larvae or well water.

### 5.3. Toxin Analyses

Hatchery water from the start of each set of bioassays was collected to test for background presence of PSTs, DSTs, and PTX2. All samples collected for the quantification of PSTs or DSTs/PTX2 were separated into two groups for extraction and analysis: dissolved and particulate toxins. Samples collected for dissolved PSTs (i.e., extracellular component of the *A. catenella* culture, initial hatchery water, and lysate) required no further extraction prior to toxin analysis. Samples for dissolved DSTs/PTX2 (i.e., extracellular component of the *D. acuminata* culture, initial hatchery water, lysate, and well water), however, were processed using solid-phase extraction (SPE) with an Oasis HLB 3-cc, 60 mg cartridge (Waters, Milford, MA, USA) prior to analysis, as described in Smith et al. [43]. 

For the analysis of particulate toxins, the sample collected for PSTs (i.e., intracellular component of *A. catenella* culture) was extracted as described in Armstrong et al. [44] with the following modification: centrifugation at 3234× *g* for 12 min at 4 °C (5804R, Eppendorf, Hauppauge, New York, NY, USA). Samples collected for particulate DSTs/PTX2 (i.e., intracellular component of *D. acuminata* culture, and oysters) were extracted with MeOH using bath sonification for 15 min at 40 kHz (M5800H, Branson, Danbury, CT, USA), or probe sonification on ice for 3 min in 30 s pulses at 40% amplitude (Branson Digital Sonifier-450, Danbury, CT, USA), respectively. Methanolic extracts for DSTs/PTX2 were centrifuged for 5 min at 3234× *g* at 4 °C (5804R, Eppendorf, Hauppauge, New York, NY, USA) and the pellet discarded.

All extracts were 0.22-µm syringe filtered (13-mm, Millex PVDF, Durapore) prior to the quantification of PSTs by hydrophilic interaction liquid chromatography (HILIC) tandem mass spectrometry (MS/MS [44,45]) or DSTs/PTX2 by ultra-performance liquid chromatography–tandem mass spectrometry with a trapping dimension and at-column dilution (UPLC–MS/MS with trap/ACD [46]). Alkaline hydrolysis was used to convert DST derivatives (i.e., esterified forms) in all DSTs/PTX2 methanolic extracts into the parent toxins OA and DTX1, following the methods of Villar-González et al. [47]. The original methanolic extracts were analyzed for PTX2 and the hydrolyzed methanolic extracts were analyzed for DSTs. Toxins analyzed included PTX2, DSTs: OA and DTX1, and PSTs: STX, NEO, GTX1, GTX2, GTX3, GTX4, GTX5, dcNEO, dcSTX, dcGTX2/3, C1, and C2. Triplicate standard curves, with 5–8 points, were run using certified reference material from NRC. All peaks with signal-to-noise ratios (S/N) < 3, or without peaks, were reported as “<DL”; peaks with S/N < 10, or S/N > 10 but with peak areas below the average of the lowest point on the standard curve, were reported as “<LOQ”.

### 5.4. Culturing

The Aquaculture Genetics and Breeding Technology Center (ABC) at the Virginia Institute of Marine Science (VIMS) cultured *P. pinguis* in batch using f/2 medium (Fritz Aquatics, Mesquite, TX, USA, [48,49]) made from hatchery water. Seven-day-old, diploid oyster larvae (*C. virginica*) were acquired from the ABC hatchery for use in the bioassays; these were spawned and raised as described in Pease et al. [42]. In the laboratory, single-cell isolate, clonal cultures of the HAB species, *A. catenella* (N5-MP3 [50]) and *D. acuminata* (DATC03; D. Anderson and M. Brosnahan, Woods Hole Oceanographic Institution, MA, USA) from the NM were acclimated step-wise to f/6-Si medium [48,49] made with autoclaved, 0.22-µm filtered seawater, salinity of 20. Batch cultures were grown at 20 °C with a 14:10 h light–dark cycle; light ranged from 38 ± 10 to 39 ± 7 *SD* µmol/m^2^/s between bioassays. Cultures of *D. acuminata* were fed live *Mesodinium rubrum* (Litostomatea, [51]). Prey were removed 24 h prior to the start of the bioassay using a 10-µm Nitex mesh; *D. acuminata* was resuspended in hatchery water. 

### 5.5. Data Analysis and Statistics

Larval inactivity and mortality data were collected as non-binomial, proportional data, i.e., *% dead* and *% inactive* at each timepoint, as the experimental design did not allow tracking of individual larvae within wells. The endpoints larval mortality and inactivity (see Section 5.2) were assessed in separate linear mixed models (LMMs) for each bioassay to examine differences between treatments. Briefly, LMMs were fitted for each bioassay with arcsine-transformed mortality or inactivity as the outcome variable, with fixed effects of treatment and time, and their interaction (when significant), and a random (intercept) effect to account for variance between wells within each treatment, i.e., well nested in treatment. First order autoregressive structure was applied to the models and models were fitted using a restricted maximum likelihood (REML) approach. Tukey’s post hoc pairwise comparisons with a Tukey–Bonferroni-adjusted alpha were used to test for differences between treatments. In the pure toxin bioassay, larval mortality and inactivity data for treatments containing PTX2 were truncated with no observations after 48 h. Larvae in these treatments were collected at 48 h for toxin analysis (see Section 5.3) due to high mortality and inactivity. PTX2 treatments were not included in pure toxin bioassay LMMs, instead, mortality and inactivity treatment effects were assessed with the Carrier control treatment using Kruskal–Wallis tests at each timepoint (24 and 48 h). Significant differences between treatments were further explored using post hoc Dunn tests with Benjamini–Hochberg-adjusted *p*-values. Statistical tests were performed in R Studio (2019) using R version 3.6.1.

## Figures and Tables

**Table 1:** Treatments within the *Alexandrium catenella* and *Dinophysis acuminata* bioassays.

Bioassay	Treatments ^a^	Cells/mL or Cells/mL Equivalent	Species
Live-Cell	Fed (Pav) ^b^	25,000	*Pavlova pinguis*
	Unfed	0	None
	Acat 10	10	*Alexandrium catenella*
	Acat 100	100	*Alexandrium catenella*
	Acat 500	500	*Alexandrium catenella*
	Acat 1000	1000	*Alexandrium catenella*
	Dacum 10	10	*Dinophysis acuminata*
	Dacum 100	100	*Dinophysis acuminata*
	Dacum 500	500	*Dinophysis acuminata*
	Dacum 1000	1000	*Dinophysis acuminata*
Lysate	Fed (Pav) ^b^	25,000	*Pavlova pinguis*
	Unfed	0	None
	Acat 100	100	*Alexandrium catenella*
	Acat 1000	1000	*Alexandrium catenella*
	Dacum 1000	1000	*Dinophysis acuminata*
	Acat 1000 × Dacum 1000	1000 *	*Alexandrium catenella* and *Dinophysis acuminata*
Pure Toxin	Carrier ^b^	0	None
	OA	10,000	None
	PTX2	10,000	None
	STX	10,000	None
	OA × PTX2	10,000	None
	OA × STX	10,000 *	None
	PTX2 × STX	10,000 *	None
	OA × PTX2 × STX	10,000 *	None

^a^ Pav = *Pavlova pinguis*, Acat = *Alexandrium catenella*, Dacum = *Dinophysis acuminata*, OA = okadaic acid, PTX2 = pectenotoxin-2, and STX = saxitoxin. ^b^ Control treatments within each bioassay. * Cells/mL equivalent of each algal species represented in the treatment, independently.

## Data Availability

The data presented in this study are contained within this article and its supplementary material.

## Supplementary Materials

The following are available online at https://www.mdpi.com/article/10.3390/toxins14050335/s1, Figure S1: Growth time series of *Alexandrium catenella* and *Dinophysis acuminata* during the live-cell bioassay, Figure S2: Toxin profiles of *Dinophysis acuminata* culture and lysate, Table S1: Least-squares means of arcsine-transformed larval inactivity in the live-cell bioassay, Table S2: Least-squares means of arcsine-transformed larval mortality in the live-cell bioassay, Table S3: Live-cell bioassay larval inactivity linear mixed effects model output, Table S4: Live-cell bioassay larval mortality linear mixed effects model output, Table S5: Least-squares means of arcsine-transformed larval inactivity in the lysate bioassay, Table S6: Least-squares means of arcsine-transformed larval mortality in the lysate bioassay, Table S7: Lysate bioassay larval inactivity linear mixed effects model output, Table S8: Lysate bioassay larval mortality linear mixed effects model output, Table S9: Pure toxin bioassay larval inactivity linear mixed effects model output, and Table S10: Pure toxin bioassay larval mortality linear mixed effects model output.
