# Supplementary material for: Effects of Two Toxin-Producing Harmful Algae, Alexandrium catenella and Dinophysis acuminata (Dinophyceae), on Activity and Mortality of Larval Shellfish
Source: Toxins (Basel). 2022 May 10;14(5):335. doi: 10.3390/toxins14050335 (PMC9143080; doi:10.3390/toxins14050335)
Supplement: Supplementary file 1 [file toxins-14-00335-s001.zip › toxins-1675547-supplementary.pdf]

toxins

Article

Effects of Two Toxin-Producing Harmful Algae, *Alexandrium catenella* and *Dinophysis acuminata* (Dinophyceae), on Activity and Mortality of Larval Shellfish

Sarah K. D. Pease, Michael L. Brosnahan, Marta P. Sanderson and Juliette L. Smith

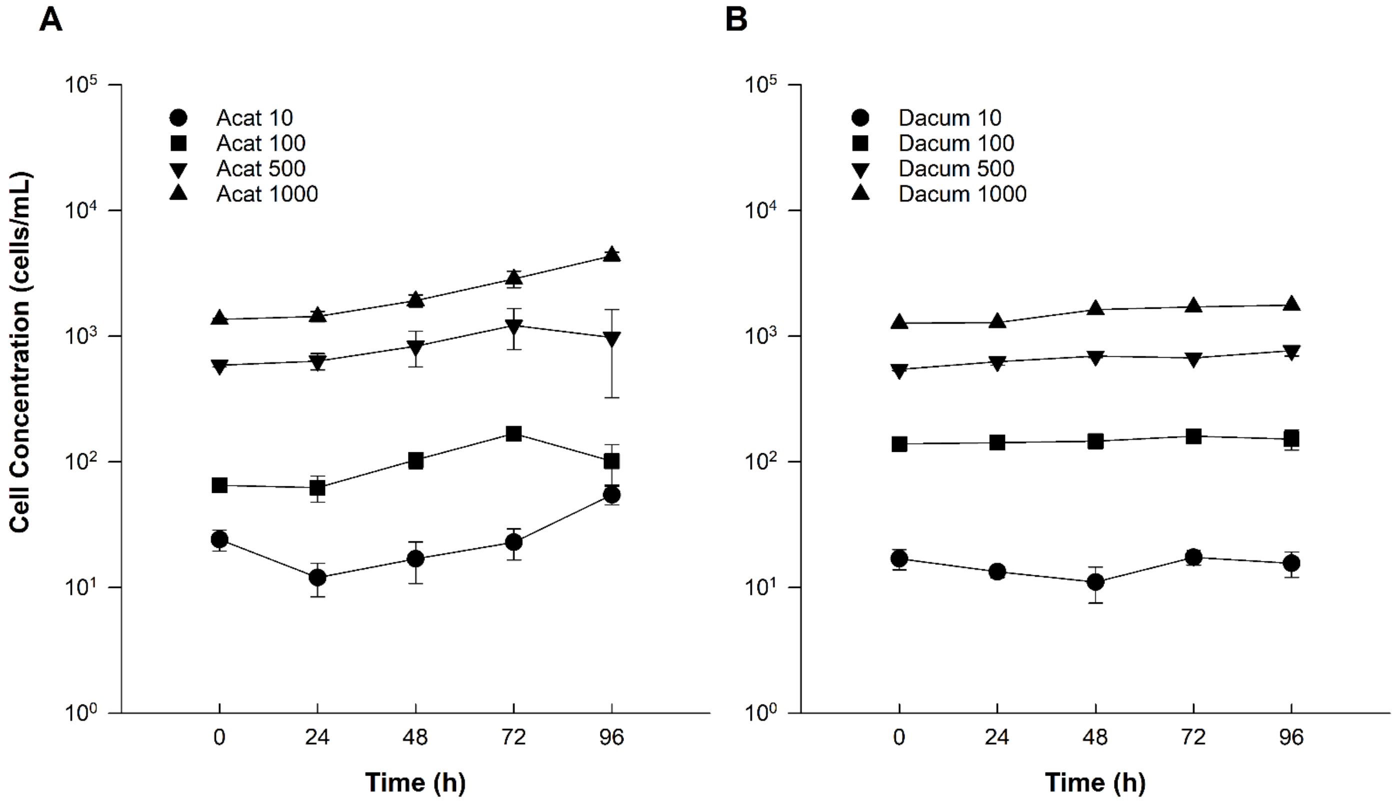

Figure S1. Growth time series of *Alexandrium catenella* and *Dinophysis acuminata* during the live-cell bioassay. **A**: *Alexandrium catenella* (Acat) and **B**: *Dinophysis acuminata* (Dacum) cell concentrations (cells/mL) over the 96-h live-cell bioassay at four different initial cell concentration treatments. Error bars show standard error (n = 3 wells per treatment).

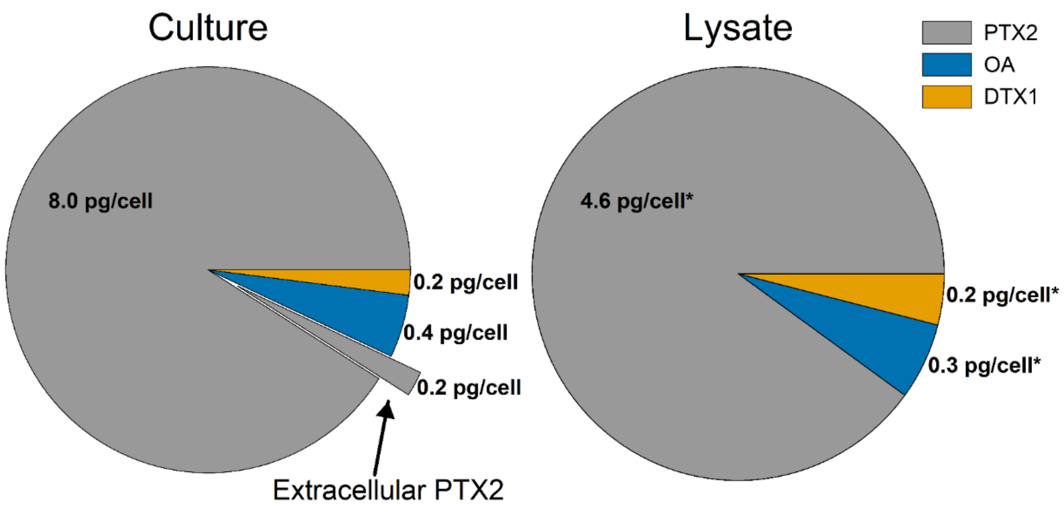

Figure S2. Toxin profiles of *Dinophysis acuminata* culture and lysate. The majority of toxins detected in the *D. acuminata* culture (strain: DATC03) were intracellular, with the exception of 0.2 pg/cell of extracellular pectenotoxin-2 (PTX2); trace amounts of okadaic acid (OA) were also detected in the extracellular fraction of the *D. acuminata* culture. Note that pg/cell is the same as ng/mL for a cell concentration of, or equivalent to, 1000 cells/mL in this study. *Lysate units are pg/cell equivalents. DTX1 = dinophysistoxin-1.

Table S1. Least-squares means of arcsine-transformed larval inactivity in the live-cell bioassay.

**Treatments***	**24 h**		**48 h**		**72 h**		**96 h**	
**Fed (Pav)**	**0.26**	**a**	**0.07**	c	0.00	f	0.27	k
Unfed	0.22	a	0.18	c	0.13	f g	0.15	k
Acat 10	0.50	a b	0.31	c d	0.24	f g h	0.85	l
Acat 100	0.34	a b	0.27	c d	0.59	g h	1.32	m
Acat 500	0.72	b	0.50	c d	1.27	i	1.40	m
Acat 1000	0.77	b	0.82	e	1.05	i j	1.32	m
Dacum 10	0.40	a b	0.24	c d	0.28	f g h	0.36	k
Dacum 100	0.20	a	0.09	c	0.18	f g h	0.18	k
Dacum 500	0.11	a	0.15	c	0.13	f g	0.18	k
Dacum 1000	0.72	b	0.65	d e	0.61	h j	0.57	k l

Standard error = 0.087 for all reported least-squares means values. Letters denote groups of treatments that were or were not significantly different from one another based on Tukey's post hoc pairwise comparisons calculated within each level of time (Tukey-Bonferroni adjusted $\alpha = 0.0125$). *Pav = *Pavlova pinguis*, Acat = *Alexandrium catenella*, Dacum = *Dinophysis acuminata*, numbers represent cell concentrations (cells/mL).

Table S2. Least-squares means of arcsine-transformed larval mortality in the live-cell bioassay.

**Treatments***	**24 h**		**48 h**		**72 h**		**96 h**	
**Fed (Pav)**	**0.00**	**a**	**0.00**	b	0.00	d	0.03	f
Unfed	0.00	a	0.00	b	0.00	d	0.00	f
Acat 10	0.00	a	0.00	b	0.00	d	0.11	f
Acat 100	0.00	a	0.00	b	0.05	d	0.17	f g
Acat 500	0.00	a	0.04	b c	0.04	d	0.39	g
Acat 1000	0.00	a	0.00	b	0.00	d	0.00	f
Dacum 10	0.00	a	0.03	b	0.09	d e	0.13	f
Dacum 100	0.00	a	0.00	b	0.03	d	0.06	f
Dacum 500	0.00	a	0.00	b	0.00	d	0.09	f
Dacum 1000	0.03	a	0.27	c	0.32	e	0.41	g

Standard error = 0.045 for all reported least-squares means values. Letters denote groups of treatments that were or were not significantly different from one another based on Tukey's post hoc pairwise comparisons calculated within each level of time (Tukey-Bonferroni adjusted α = 0.0125). *Pav = *Pavlova pinguis*, Acat = *Alexandrium catenella*, Dacum = *Dinophysis acuminata*, numbers represent cell concentrations (cells/mL).

Table S3. Live-cell bioassay larval inactivity linear mixed effects model output. .

**Type III Tests of Fixed Effects**				
	**Numerator *df***	**Denominator *df***	***F*-value**	***p*-value**
Intercept	1	270	513	< 0.0001
Time	3	270	43	< 0.0001
Treatment	9	90	25	< 0.0001
Time:Treatment	27	270	9	< 0.0001

**Random Effects Variance Components Estimates**		
	**Variance**	***SD***
Well (Intercept)	0.026	0.16
Residual	0.050	0.22

**Estimates of Fixed Effects**								
		**Estimate**	***SE***	***df***	***t*-value**	***p*-value**	***CI* Lower**	***CI* Upper**
Intercept		0.26	0.087	270	3.0	0.0030	0.089	0.43
Time	24	0	0	-	-	-	-	-
	48	-0.19	0.083	270	-2.3	0.023	-0.35	-0.026
	72	-0.26	0.095	270	-2.7	0.0066	-0.45	-0.073
	96	0.0061	0.098	270	0.062	0.95	-0.19	0.20
Treatment*	Fed (Pav)	0	0	-	-	-	-	-
	Unfed	-0.037	0.12	90	-0.30	0.77	-0.28	0.21
	Acat 10	0.24	0.12	90	2.0	0.053	-0.0027	0.49
	Acat 100	0.075	0.12	90	0.61	0.54	-0.17	0.32
	Acat 500	0.46	0.12	90	3.8	0.0003	0.22	0.71
	Acat 1000	0.51	0.12	90	4.1	0.0001	0.26	0.75
	Dacum 10	0.14	0.12	90	1.1	0.27	-0.11	0.38
	Dacum 100	-0.056	0.12	90	-0.46	0.65	-0.30	0.19
	Dacum 500	-0.15	0.12	90	-1.2	0.23	-0.39	0.095
	Dacum 1000	0.47	0.12	90	3.8	0.0002	0.23	0.72
Time:Treatment	24:Fed (Pav)	0	0	-	-	-	-	-
	48:Fed (Pav)	0	0	-	-	-	-	-
	72:Fed (Pav)	0	0	-	-	-	-	-
	96:Fed (Pav)	0	0	-	-	-	-	-
	24:Unfed	0	0	-	-	-	-	-
	48:Unfed	0.15	0.12	270	1.2	0.22	-0.086	0.38
	72:Unfed	0.17	0.13	270	1.3	0.21	-0.094	0.43
	96:Unfed	-0.077	0.14	270	-0.56	0.58	-0.35	0.20
	24:Acat 10	0	0	-	-	-	-	-
	48:Acat 10	-0.0020	0.12	270	-0.017	0.99	-0.23	0.23

72:Acat 10	-0.0033	0.13	270	-0.025	0.98	-0.27	0.26
	**Estimate**	***SE***	***df***	***t*-value**	***p*-value**	***CI* Lower**	***CI* Upper**
96:Acat 10	0.35	0.14	270	2.5	0.014	0.072	0.62
24:Acat 100	0	0	-	-	-	-	-
48:Acat 100	0.12	0.12	270	1.0	0.30	-0.11	0.35
72:Acat 100	0.51	0.13	270	3.8	0.0002	0.25	0.78
96:Acat 100	0.98	0.14	270	7.1	0	0.71	1.3
24:Acat 500	0	0	-	-	-	-	-
48:Acat 500	-0.035	0.12	270	-0.30	0.77	-0.27	0.20
72:Acat 500	0.80	0.13	270	6.0	0	0.54	1.1
96:Acat 500	0.67	0.14	270	4.8	0	0.40	0.95
24:Acat 1000	0	0	-	-	-	-	-
48:Acat 1000	0.24	0.12	270	2.1	0.038	0.013	0.48
72:Acat 1000	0.54	0.13	270	4.1	0.0001	0.28	0.81
96:Acat 1000	0.55	0.14	270	3.9	0.0001	0.27	0.82
24:Dacum 10	0	0	-	-	-	-	-
48:Dacum 10	0.029	0.12	270	0.25	0.80	-0.20	0.26
72:Dacum 10	0.14	0.13	270	1.1	0.29	-0.12	0.41
96:Dacum 10	-0.041	0.14	270	-0.30	0.77	-0.31	0.23
24:Dacum 100	0	0	-	-	-	-	-
48:Dacum 100	0.073	0.12	270	0.62	0.54	-0.16	0.30
72:Dacum 100	0.23	0.13	270	1.7	0.082	-0.030	0.50
96:Dacum 100	-0.027	0.14	270	-0.19	0.85	-0.30	0.25
24:Dacum 500	0	0	-	-	-	-	-
48:Dacum 500	0.23	0.12	270	2.0	0.050	0.0004	0.46
72:Dacum 500	0.28	0.13	270	2.1	0.036	0.019	0.55
96:Dacum 500	0.066	0.14	270	0.48	0.63	-0.21	0.34
24:Dacum 1000	0	0	-	-	-	-	-
48:Dacum 1000	0.11	0.12	270	0.96	0.34	-0.12	0.34
72:Dacum 1000	0.14	0.13	270	1.0	0.30	-0.12	0.40
96:Dacum 1000	-0.17	0.14	270	-1.2	0.22	-0.45	0.10

Outcome variable = arcsine-transformed larval inactivity including mortality. *df* = degrees of freedom, *SD* = standard deviation, *SE* = standard error, *CI* = 95% confidence interval. *Pav = *Pavlova pinguis*, Acat = *Alexandrium catenella*, Dacum = *Dinophysis acuminata*, numbers represent cell concentrations (cells/mL).

Table S4. Live-cell bioassay larval mortality linear mixed effects model output. .

Type III Tests of Fixed Effects

	Numerator *df*	Denominator *df*	*F*-value	*p*-value
Intercept	1	270	35	< 0.0001
Time	3	270	24	< 0.0001
Treatment	9	90	5	< 0.0001
Time:Treatment	27	270	4	< 0.0001

Random Effects Variance Components Estimates

	Variance	*SD*
Well (Intercept)	0.020	0.14
Residual	$1.1E^{-10}$	$1.1E^{-5}$

Estimates of Fixed Effects

		Estimate	*SE*	*df*	*t*-value	*p*-value	*CI* Lower	*CI* Upper
Intercept		0	0.045	270	0	1	-0.088	0.088
Time	24	0	0	-	-	-	-	-
	48	0	0.040	270	0	1	-0.079	0.079
	72	0	0.051	270	0	1	-0.10	0.10
	96	0.031	0.056	270	0.54	0.59	-0.080	0.14
Treatment*	Fed (Pav)	0	0	-	-	-	-	-
	Unfed	0	0.064	90	0	1	-0.13	0.13
	Acat 10	0	0.064	90	0	1	-0.13	0.13
	Acat 100	0	0.064	90	0	1	-0.13	0.13
	Acat 500	0	0.064	90	0	1	-0.13	0.13
	Acat 1000	0	0.064	90	0	1	-0.13	0.13
	Dacum 10	0	0.064	90	0	1	-0.13	0.13
	Dacum 100	0	0.064	90	0	1	-0.13	0.13
	Dacum 500	0	0.064	90	0	1	-0.13	0.13
	Dacum 1000	0.032	0.064	90	0.51	0.61	-0.094	0.16
Time:Treatment	24:Fed (Pav)	0	0	-	-	-	-	-
	48:Fed (Pav)	0	0	-	-	-	-	-
	72:Fed (Pav)	0	0	-	-	-	-	-
	96:Fed (Pav)	0	0	-	-	-	-	-
	24:Unfed	0	0	-	-	-	-	-
	48:Unfed	0	0.057	270	0	1	-0.11	0.11
	72:Unfed	0	0.072	270	0	1	-0.14	0.14
	96:Unfed	-0.031	0.080	270	-0.39	0.70	-0.19	0.13
	24:Acat 10	0	0	-	-	-	-	-
	48:Acat 10	0	0.057	270	0	1	-0.11	0.11

72:Acat 10	0	0.072	270	0	1	-0.14	0.14
	**Estimate**	***SE***	***df***	***t*-value**	***p*-value**	***CI* Lower**	***CI* Upper**
96:Acat 10	0.077	0.080	270	0.97	0.33	-0.079	0.23
24:Acat 100	0	0	-	-	-	-	-
48:Acat 100	0	0.057	270	0	1	-0.11	0.11
72:Acat 100	0.052	0.072	270	0.73	0.47	-0.089	0.19
96:Acat 100	0.14	0.080	270	1.8	0.071	-0.012	0.30
24:Acat 500	0	0	-	-	-	-	-
48:Acat 500	0.036	0.057	270	0.64	0.53	-0.076	0.15
72:Acat 500	0.036	0.072	270	0.50	0.62	-0.11	0.18
96:Acat 500	0.36	0.080	270	4.5	0	0.20	0.52
24:Acat 1000	0	0	-	-	-	-	-
48:Acat 1000	0	0.057	270	0	1	-0.11	0.11
72:Acat 1000	0	0.072	270	0	1	-0.14	0.14
96:Acat 1000	-0.031	0.080	270	-0.39	0.70	-0.19	0.13
24:Dacum 10	0	0	-	-	-	-	-
48:Dacum 10	0.029	0.057	270	0.52	0.61	-0.083	0.14
72:Dacum 10	0.094	0.072	270	1.3	0.19	-0.048	0.24
96:Dacum 10	0.095	0.080	270	1.2	0.23	-0.061	0.25
24:Dacum 100	0	0	-	-	-	-	-
48:Dacum 100	0	0.057	270	0	1	-0.11	0.11
72:Dacum 100	0.034	0.072	270	0.47	0.64	-0.11	0.18
96:Dacum 100	0.034	0.080	270	0.43	0.67	-0.12	0.19
24:Dacum 500	0	0	-	-	-	-	-
48:Dacum 500	0	0.057	270	0	1	-0.11	0.11
72:Dacum 500	0	0.072	270	0	1	-0.14	0.14
96:Dacum 500	0.063	0.080	270	0.79	0.43	-0.094	0.22
24:Dacum 1000	0	0	-	-	-	-	-
48:Dacum 1000	0.24	0.057	270	4.2	0	0.13	0.35
72:Dacum 1000	0.29	0.072	270	4.0	0.0001	0.14	0.43
96:Dacum 1000	0.34	0.080	270	4.3	0	0.19	0.50

Outcome variable = arcsine-transformed larval mortality. *df* = degrees of freedom, *SD* = standard deviation, *SE* = standard error, *CI* = 95% confidence interval. *Pav = *Pavlova pinguis*, Acat = *Alexandrium catenella*, Dacum = *Dinophysis acuminata*, numbers represent cell concentrations (cells/mL).

Table S5. Least-squares means of arcsine-transformed larval inactivity in the lysate bioassay.

**Treatments***	**24 h**		**48 h**		**72 h**		**96 h**	
Fed (Pav)	0.03	a	0.06	c d	0.07	f g	0.06	i j
Unfed	0.06	a	0.09	c d	0.13	f g	0.10	i j
Acat 100	0.03	a	0.00	d	0.00	g	0.00	j
Acat 1000	0.21	a b	0.16	c d e	0.14	f g	0.13	i j
Dacum 1000	0.34	b	0.38	e	0.51	h	0.59	k
Acat 1000 × Dacum 1000	0.11	a b	0.33	c e	0.28	f h	0.29	i

Standard error = 0.078 for all reported least-squares means values. Letters denote groups of treatments that were or were not significantly different from one another based on Tukey's post hoc pairwise comparisons calculated within each level of time (Tukey-Bonferroni adjusted $\alpha = 0.0125$). *Pav = *Pavlova pinguis*, Acat = *Alexandrium catenella*, Dacum = *Dinophysis acuminata*, numbers represent cell concentration equivalents (cells/mL equiv.).

Table S6. Least-squares means of arcsine-transformed larval mortality in the lysate bioassay.

**Treatments***	**24 h**		**48 h**		**72 h**		**96 h**	
Fed (Pav)	0.00	a	0.00	b	0.00	c	0.00	d
Unfed	0.00	a	0.03	b	0.06	c	0.06	d
Acat 100	0.00	a	0.00	b	0.00	c	0.00	d
Acat 1000	0.00	a	0.00	b	0.00	c	0.03	d e
Dacum 1000	0.00	a	0.07	b	0.16	c	0.23	f
Acat 1000 × Dacum 1000	0.00	a	0.10	b	0.15	c	0.18	e f

Standard error = 0.034 for all reported least-squares means values. Letters denote groups of treatments that were or were not significantly different from one another based on Tukey's post hoc pairwise comparisons calculated within each level of time (Tukey-Bonferroni adjusted $\alpha = 0.0125$). *Pav = *Pavlova pinguis*, Acat = *Alexandrium catenella*, Dacum = *Dinophysis acuminata*, numbers represent cell concentration equivalents (cells/mL equiv.).

Table S7. Lysate bioassay larval inactivity linear mixed effects model output. .

**Type III Tests of Fixed Effects**				
	**Numerator *df***	**Denominator *df***	***F*-value**	***p*-value**
Intercept	1	162	102	< 0.0001
Time	3	162	2	0.07
Treatment	5	54	16	< 0.0001
Time:Treatment	15	162	2	0.01

**Random Effects Variance Components Estimates**		
	**Variance**	***SD***
Well (Intercept)	0.0099	0.099
Residual	0.020	0.14

**Estimates of Fixed Effects**								
		**Estimate**	***SE***	***df***	***t*-value**	***p*-value**	***CI* Lower**	***CI* Upper**
Intercept		0.031	0.055	162	0.56	0.58	-0.078	0.14
Time	24	0	0	-	-	-	-	-
	48	0.034	0.055	162	0.61	0.54	-0.075	0.14
	72	0.036	0.061	162	0.59	0.56	-0.086	0.16
	96	0.034	0.063	162	0.54	0.59	-0.091	0.16
Treatment*	Fed (Pav)	0	0	-	-	-	-	-
	Unfed	0.034	0.077	54	0.44	0.67	-0.12	0.19
	Acat 100	0	0.077	54	0	1	-0.16	0.16
	Acat 1000	0.17	0.077	54	2.3	0.028	0.019	0.33
	Dacum 1,000	0.31	0.077	54	4.1	0.0002	0.16	0.47
	Acat 1000 × Dacum 1000	0.084	0.077	54	1.1	0.28	-0.080	0.24
Time:Treatment	24:Fed (Pav)	0	0	-	-	-	-	-
	48:Fed (Pav)	0	0	-	-	-	-	-
	72:Fed (Pav)	0	0	-	-	-	-	-
	96:Fed (Pav)	0	0	-	-	-	-	-
	24:Unfed	0	0	-	-	-	-	-
	48:Unfed	-0.0082	0.078	162	-0.10	0.92	-0.16	0.15
	72:Unfed	0.031	0.087	162	0.35	0.73	-0.14	0.20
	96:Unfed	-0.0034	0.089	162	-0.038	0.97	-0.18	0.17
	24:Acat 100	0	0	-	-	-	-	-
	48:Acat 100	-0.065	0.078	162	-0.83	0.41	-0.22	0.090
	72:Acat 100	-0.067	0.087	162	-0.77	0.44	-0.24	0.11
	96:Acat 100	-0.065	0.089	162	-0.72	0.47	-0.24	0.11

	Estimate	*SE*	*df*	*t*-value	*p*-value	*CI* Lower	*CI* Upper
24:Acat 1000	0	0	-	-	-	-	-
48:Acat 1000	-0.079	0.078	162	-1.0	0.31	-0.23	0.075
72:Acat 1000	-0.010	0.087	162	-1.1	0.25	-0.27	0.072
96:Acat 1000	-0.11	0.089	162	-1.2	0.23	-0.28	0.068
24:Dacum 1000	0	0	-	-	-	-	-
48:Dacum 1000	0.0026	0.078	162	0.033	0.97	-0.15	0.16
72:Dacum 1000	0.13	0.087	162	1.5	0.14	-0.042	0.30
96:Dacum 1000	0.21	0.089	162	2.4	0.019	0.036	0.39
24:Acat 1000 × Dacum 1000	0	0	-	-	-	-	-
48: Acat 1000 × Dacum 1000	0.18	0.078	162	2.3	0.021	0.028	0.34
72: Acat 1000 × Dacum 1000	0.13	0.087	162	1.5	0.13	-0.038	0.31
96: Acat 1000 × Dacum 1000	0.14	0.089	162	1.6	0.12	-0.036	0.32

Outcome variable = arcsine-transformed larval inactivity including mortality. *df* = degrees of freedom, *SD* = standard deviation, *SE* = standard error, *CI* = 95% confidence interval. *Pav = *Pavlova pinguis*, Acat = *Alexandrium catenella*, Dacum = *Dinophysis acuminata*, numbers represent cell concentrations (cells/mL).

Table S8. Lysate bioassay larval mortality linear mixed effects model output. .

**Type III Tests of Fixed Effects**				
	**Numerator *df***	**Denominator *df***	***F*-value**	***p*-value**
Intercept	1	162	16	< 0.0001
Time	3	162	8	< 0.0001
Treatment	5	54	4	0.007
Time:Treatment	15	162	2	0.01

**Random Effects Variance Components Estimates**		
	**Variance**	***SD***
Well (Intercept)	$1.6E^{-11}$	$4.0E^{-6}$
Residual	0.012	0.11

**Estimates of Fixed Effects**								
		**Estimate**	***SE***	***df***	***t*-value**	***p*-value**	***CI* Lower**	***CI* Upper**
Intercept		0	0.034	162	0	1	-0.067	0.067
Time	24	0	0	-	-	-	-	-
	48	0	0.029	162	0	1	-0.056	0.056
	72	0	0.037	162	0	1	-0.072	0.072
	96	0	0.041	162	0	1	-0.081	0.081
Treatment*	Fed (Pav)	0	0	-	-	-	-	-
	Unfed	0	0.048	54	0	1	-0.097	0.097
	Acat 100	0	0.048	54	0	1	-0.097	0.097
	Acat 1000	0	0.048	54	0	1	-0.097	0.097
	Dacum 1000	0	0.048	54	0	1	-0.097	0.097
	Acat 1000 × Dacum 1000	0	0.048	54	0	1	-0.097	0.097
Time:Treatment	24:Fed (Pav)	0	0	-	-	-	-	-
	48:Fed (Pav)	0	0	-	-	-	-	-
	72:Fed (Pav)	0	0	-	-	-	-	-
	96:Fed (Pav)	0	0	-	-	-	-	-
	24:Unfed	0	0	-	-	-	-	-
	48:Unfed	0.032	0.040	162	0.80	0.43	-0.048	0.11
	72:Unfed	0.064	0.052	162	1.2	0.22	-0.038	0.17
	96:Unfed	0.064	0.058	162	1.1	0.27	-0.051	0.18
	24:Acat 100	0	0	-	-	-	-	-
	48:Acat 100	0	0.040	162	0	1	-0.080	0.080
	72:Acat 100	0	0.052	162	0	1	-0.10	0.10
	96:Acat 100	0	0.058	162	0	1	-0.11	0.11

	Estimate	*SE*	*df*	*t*-value	*p*-value	*CI* Lower	*CI* Upper
24:Acat 1000	0	0	-	-	-	-	-
48:Acat 1000	0	0.040	162	0	1	-0.080	0.080
72:Acat 1000	0	0.052	162	0	1	-0.10	0.10
96:Acat 1000	0.031	0.058	162	0.53	0.60	-0.084	0.15
24:Dacum 1000	0	0	-	-	-	-	-
48:Dacum 1000	0.066	0.040	162	1.6	0.10	-0.014	0.15
72:Dacum 1000	0.16	0.052	162	3.1	0.0024	0.057	0.26
96:Dacum 1000	0.23	0.058	162	3.9	0.0001	0.11	0.34
24:Acat 1000 × Dacum 1000	0	0	-	-	-	-	-
48: Acat 1000 × Dacum 1000	0.098	0.040	162	2.4	0.016	0.019	0.18
72: Acat 1000 × Dacum 1000	0.15	0.052	162	2.8	0.0054	0.044	0.25
96: Acat 1000 × Dacum 1000	0.18	0.058	162	3.1	0.0025	0.064	0.29

Outcome variable = arcsine-transformed larval mortality. *df* = degrees of freedom, *SD* = standard deviation, *SE* = standard error, *CI* = 95% confidence interval. *Pav = *Pavlova pinguis*, Acat = *Alexandrium catenella*, Dacum = *Dinophysis acuminata*, numbers represent cell concentrations (cells/mL).

Table S9. Pure toxin bioassay larval inactivity linear mixed effects model output. .

**Type III Tests of Fixed Effects**				
	**Numerator *df***	**Denominator *df***	***F*-value**	***p*-value**
Intercept	1	117	21	< 0.0001
Time	3	117	0.4	0.7
Treatment	3	36	0.5	0.7

**Random Effects Variance Components Estimates**		
	**Variance**	***SD***
Well (Intercept)	0.0013	0.036
Residual	0.021	0.14

**Estimates of Fixed Effects**								
		**Estimate**	***SE***	***df***	***t*-value**	***p*-value**	***CI* Lower**	***CI* Upper**
Intercept		0.064	0.038	117	1.7	0.098	-0.012	0.14
Time	24	0	0	-	-	-	-	-
	48	-0.0092	0.022	117	-0.42	0.68	-0.052	0.034
	72	-0.0092	0.027	117	-0.34	0.74	-0.063	0.044
	96	0.014	0.029	117	0.49	0.63	-0.044	0.073
Treatment*	Carrier	0	0	-	-	-	-	-
	OA	-0.013	0.049	36	-0.25	0.80	-0.11	0.088
	STX	0.040	0.049	36	0.80	0.43	-0.060	0.14
	OA × STX	0.028	0.059	36	0.57	0.57	-0.072	0.13

Outcome variable = arcsine-transformed larval inactivity including mortality. *df* = degrees of freedom, *SD* = standard deviation, *SE* = standard error, *CI* = 95% confidence interval. *Carrier = carrier control (4% MeOH and 3 μM HCl), OA = okadaic acid, STX = saxitoxin.

Table S10. Pure toxin bioassay larval mortality linear mixed effects model output. .

**Type III Tests of Fixed Effects**				
	**Numerator *df***	**Denominator *df***	***F*-value**	***p*-value**
Intercept	1	117	5	0.04
Time	3	117	4	0.02
Treatment	3	36	0.2	0.9

**Random Effects Variance Components Estimates**		
	**Variance**	***SD***
Well (Intercept)	$1.4E^{-12}$	$1.2E^{-6}$
Residual	0.0045	0.067

**Estimates of Fixed Effects**								
		**Estimate**	***SE***	***df***	***t*-value**	***p*-value**	***CI* Lower**	***CI* Upper**
Intercept		-0.000065	0.017	117	-0.0038	1.0	-0.034	0.033
Time	24	0	0	-	-	-	-	-
	48	0	0.011	117	0	1	-0.021	0.021
	72	0.016	0.013	117	1.2	0.22	-0.010	0.042
	96	0.04	0.014	117	2.9	0.0049	0.012	0.068
Treatment*	Carrier	0	0	-	-	-	-	-
	OA	-0.0053	0.021	36	-0.25	0.81	-0.049	0.038
	STX	-0.0053	0.021	36	-0.25	0.81	-0.049	0.038
	OA × STX	0.011	0.021	36	0.50	0.62	-0.033	0.054

Outcome variable = arcsine-transformed larval mortality. *df* = degrees of freedom, *SD* = standard deviation, *SE* = standard error, *CI* = 95% confidence interval. *Carrier = carrier control (4% MeOH and 3 μM HCl), OA = okadaic acid, STX = saxitoxin.
